# Parliament and the Profession

**Published:** 1916-07-22

**Authors:** 


### PARLIAMENT AND THE PROFESSION.

### Dental Treatment of Troops.

Mr. Forster again asserted that all applications for dental surgeons with our troops at home and abroad had been fully and promptly met. There was a large waiting list of candidates for dental commissions, and each application was considered in the order in which it was received, preference being given to candidates who had voluntarily enlisted in the Army.

### Sergeant Clerks in the R.A.M.C.

Asked by Mr. E. Harvey if the pay of a sergeant clerk in the R.A.M.C. was higher than that of one in the Territorial Force when serving abroad and doing exactly similar work, Mr. Forster stated that the special rates given to certain clerks who belonged to the peace establishment here had not been extended to clerks of the Territorial Force. The question as to how many clerks should receive these special rates was still under consideration.

### Haslar Hospital Comforts.

Mr. R. Gwynne having expressed a doubt that the arrangements for the comfort and entertainment of patients at Haslar Naval Hospital were adequate, the Parliamentary Secretary to the Admiralty stated that although smoking was not allowed in the wards, there were two large smoking sheds and six large colonnades where, as well as in the airy grounds, smoking was permitted. Patients were allowed to sit and lie on the grass in the grounds, and for them were available easy-chairs, folding chairs, and ordinary bath-chairs, while numerous iron garden seats and forms were provided in the grounds. Further, said Dr. Macnamara, entertainments were given on five afternoons each week in the Erroll Hall.

### Voluntary Ambulance Drivers and Military Service.

The voluntary ambulance column attached to the London district has, according to Mr. Joynson Hicks, dealt with over 100,000 cases at no cost to the State, but is on the verge of collapse owing to the drivers being conscripted. He asked what steps the War Office proposed to take to ensure the safe moving of the wounded. Mr. Forster, who replied, said he was not aware the column was on the verge of collapse, and suggested that those who controlled the organisation should submit a list of the drivers who were employed on the work of the ambulance column. He could not, however, undertake that this body should be treated differently from the British Red Cross Society or the Royal Army Medical Corps as regards the enforcement of the liability to general service.

### Nerve-shaken Soldiers.

The definite statement was made by Mr. Forster that no uncertifiable soldiers suffering from nerve strain were treated in any other institutions than military hospitals under the control of the War Office. There were 229 cases in the Springfield War Hospital, these being isolated from the rest of the hospital, and treated by officers in the employ of the Army Medical Service. There are now 320 soldiers in the County of Middlesex War Hospital, Napsbury, all of whom were certifiable when sent there. A limited number of cases which, after a period of from four to six months' observation and treatment, are considered to be incurable, have been discharged to asylums. The number is 108. Replying to a question respecting the treatment of nerve-shaken sailors, Dr. Macnamara stated that men invalided from the Navy for insanity attributable to the Service, and in non-attributable cases those with length of service of ten years and upwards to their credit, were, irrespective of the cause of insanity, sent to Yarmouth Hospital.

### Accommodation for the Mentally Deficient.

In a statement in reply to Major Astor, the Home Secretary gave particulars as to the provision made by local authorities for the treatment of the mentally deficient under the Mental Deficiency Act, 1913. The total number of local authorities under the Act is 125, and the number which have submitted one or more schemes for giving effect to the Act is eighty-seven. Other authorities have been urged to carry out the important but comparatively inexpensive duties of ascertaining the numbers of defectives for whom they are responsible, and of providing supervision and guardianship where required. The restriction of capital issues during the war prevents local authorities from purchasing land or buildings with a view to the provision of institutional accommodation on an important scale, but the Board of Control has suggested that they should provide for urgent cases by leasing or adapting existing buildings where this can be done at little cost, or entering into contracts with the managers of existing institutions or applying for the approval of suitable portions of Poor-Law premises.

### Other Matters of Interest.

R.A.M.C. Captains.—There are 254 Regular captains in the R.A.M.C. who have held that rank since the war began. In giving this information Mr. Forster added that it was not proposed to make any permanent promotions in the near future.

Surgeon-General Sir W. Babtie.—Replying to Colonel Yate, Mr. Chamberlain stated that General Babtie left India for Egypt on February 6, and returned on March 19. He was deputed to Egypt at the instance of the War Office to report on the Indian medical arrangements there. During his absence the routine duties of the office were carried on by the Deputy-Director of Medical Services. The Government of India report that the latter dealt with no questions of policy.

